# The Effects of Sildenafil and/or Nitroglycerin on Random-pattern Skin Flaps After Nicotine Application in Rats

**DOI:** 10.1038/s41598-020-60128-w

**Published:** 2020-02-21

**Authors:** Mohamed A. Ellabban, Islam Omar Abdel Fattah, Ghada Abdel Kader, Omar Salah Eldeen, Amir E. Mehana, Dina M. Khodeer, Hossam Hosny, Mahmoud S. Elbasiouny, Suhail Masadeh

**Affiliations:** 1 Plastic and Reconstructive Surgery Unit, Suez Canal University Hospitals and Medical School, Ismailia, Egypt; 2 Human Anatomy and Embryology Department, Faculty of Medicine, Suez Canal University, Ismailia, Egypt; 3 Department of Zoology, Faculty of Science, Suez Canal University, Ismailia, Egypt; 4 Department of Pharmacology and Toxicology, Faculty of Pharmacy, Suez Canal University, Ismailia, Egypt; 5 Plastic and Reconstructive Surgery Department, Cairo University, Giza, Egypt; 6 Plastic Surgery Unit National Laser Institute, Cairo University, Giza, Egypt; 7 Podiatric Surgery, UC Heath, Cincinnati, USA

**Keywords:** Histology, Clinical pharmacology, Zoology, Skin diseases, Drug development

## Abstract

Smoking aggravates skin necrosis as a complication of random-pattern flap ischaemia. Sildenafil and nitroglycerin (NTG) are vasodilator agents that may affect skin flap survival. Fifty rats were subjected to a dorsal random-pattern flap operation and randomly divided into 5 groups. The control group received no treatment. The ischaemic group were administered local nicotine injections. The sildenafil group were administered oral sildenafil treatment in addition to the same intervention as the ischaemic group. The NTG group received topical NTG ointment application instead of sildenafil. The combined group were given both sildenafil and NTG treatments. After 7 days, all rats were sacrificed for flap assessment. Flap survival percentages at the 3^rd^ and 7^th^ days were significantly higher in the combined group than in the other study groups. Histologically, the ischaemic group exhibited dermal disorganization and inflammatory cell infiltration, which were improved in the 3 treated groups; however, the combined group presented the most relevant effect. The epidermal thickness showed a decrease in the ischaemic group (23.1 μm) that was significantly increased in the sildenafil (28.4 μm), NTG (28.8 μm) and combined (35.8 μm) groups. Immunohistochemically, the combined group exhibited a significant decrease in the apoptotic index and an increase in the proliferative index (2.3 and 56.9%, respectively) compared to those in the ischaemic (63.2 and 3%), sildenafil (41.7 and 28.1%) and NTG (39.3 and 30.4%) groups. Transmission electron microscopy (TEM) showed that the combined group displayed improvement in most of the ischaemic changes. Our analyses suggest that the combined use of sildenafil and NTG is more efficacious than using only one of these treatments for skin flap survival.

## Introduction

Random skin flaps are widely used options for the reconstruction of large acquired or congenital skin defects^1^. Survival of these flaps is highly reliant on oxygen delivery to their tissues^2^. The main challenge is that the distal parts of the flap suffer from a reduced blood supply, which can trigger skin necrosis^3^. This reduction in the blood supply is mainly caused by anatomical or haemodynamic factors^4^. During vascular regeneration, ischaemia/reperfusion injury occurs, promoting oxidative stress and apoptosis^3^. Additionally, other factors, such as inadequate angiogenesis and inflammatory reactions, play roles in the pathogenesis of flap necrosis^5^.

Skin flap ischaemic necrosis and increased incidence of infection can be aggravated by nicotine, which endangers the results of plastic skin reconstruction^6^. Smoking increases the risk of skin flap complications, which has been observed in clinical and experimental studies in both rats and hamsters^7^. Smokers have a higher risk of developing skin flap necrosis, namely, 13 times higher than that of non-smokers, which can result in flap loss or secondary contractures that negatively affect the aesthetic outcomes^6,8^. The potential mechanisms of this increased risk in smokers are highly complicated and may include decreased flap perfusion due to vasoconstriction, increased tissue reactive oxygen species, direct effects of smoke ingredients on the efficacy of the cellular healing mechanisms and indirect effects of smoking, such as the impaired release of endothelial nitric oxide (NO), which may accompany the resultant vascular deficiency. Most of those factors are attributed to the nicotinic effect^9^.

Recent publications have been directed towards improving the skin flap survival rate by increasing tissue perfusion and oxygenation^2^. One of the therapeutic agents that have numerous actions in various diseases is sildenafil^10^. Since the 1980s, sildenafil has been used as an antianginal drug, as well as to treat erectile dysfunction in males^11^. It is also known that sildenafil has positive effects on tissue healing through a direct effect on endothelial cell function and cellular apoptosis^12^. Furthermore, the drug stimulates vasodilatation by inducing vascular smooth muscle relaxation. Although the capillaries do not have vascular smooth muscle, they passively will dilate based on amount of blood flow coming from the arteriole and hence recovering microcirculatory tissue perfusion. Sildenafil can inhibit platelet aggregation, preventing small vessel obstruction^13^.

Nitroglycerin (NTG) ointment is safe, inexpensive, and easy to apply and has a rapid onset of action. Despite all of its advantages, NTG is not commonly used for the prevention of skin flap necrosis. This may be due to the controversy over its beneficial effect in promoting skin flap survival^14,15^. For example, in laboratory animal experiments, NTG was shown to have both positive and negative results in the enhancement of skin flap viability^15^, although it has been proven that NTG ointment has a local vasodilator effect on large capacitance vessels and thus may increase skin flap survival rates^14,16^.

Hence, in this research, we tested the ability of sildenafil and NTG alone or in combination to improve distal cutaneous flap survival in rats.

## Materials and Methods

### Animals

A total of fifty Sprague-Dawley male rats weighing 175 ± 25 g. were purchased from the Faculty of Veterinary Medicine, Suez Canal University, Ismailia, Egypt. The rats were kept in an air-conditioned room with a 12-hour light/dark cycle and were provided with tap water and rodent pellets *ad libitum*. All experimental procedures were performed in accordance with the guidelines of the National Institutes of Health (NIH) for the care and use of laboratory animals. Experimental design and animal handling were approved by the Research Ethical Committee of the Faculty of Pharmacy, Suez Canal University, Ismailia, Egypt (the approval no 201807RA1). All efforts were made to minimize animal suffering.

### Experimental design

After 2 weeks for acclimatization, rats were randomly divided into 5 equal groups (n = 10 per group) as follows:

Group I (control group): rats were subjected to surgical procedures involving dorsal random-pattern skin flaps in the form of flap elevation followed by resuturing and received subcutaneous (SC) injections of normal saline locally at the base of the future flap for 40 days before surgery and 7 days after with no nicotine treatment^17^.

Group II (ischaemic group): rats were subjected to the same surgical procedures as the control group and additionally received daily SC of nicotine locally at the base of the future flap for 40 days before surgery and 7 days after the operation^18^.

Group III (sildenafil group): rats received oral sildenafil treatment for 7 days after the flap operation in addition to daily SC nicotine injections locally at the base of the future flap for 40 days before and 7 days after the random-pattern skin flap surgery^19^.

Group IV (NTG group): rats received local NTG ointment treatment for 7 days after the flap operation in addition to daily SC nicotine injections locally at the base of the future flap for 40 days before and 7 days after the random-pattern skin flap surgery^17^.

Group V (combined group): rats received combined treatment of oral sildenafil and local NTG for 7 days after the flap operation in addition to daily nicotine administration by SC injections locally at the base of the future flap for 40 days before and 7 days after the the surgical procedures for the random-pattern skin flap^17^.

### Skin flap surgery

After induction of general anaesthesia by intraperitoneal (IP) injection of 60 mg/kg ketamine and 10 mg/kg xylazine, the rats were placed in the prone position, and the hair of the back was shaved using electric clippers. The skin was then disinfected by povidone-iodine solution. A transparent template of 9 × 2 cm was used to mark the flap area by using a permanent pen marker (Supplementary Fig. 1A,B). A caudally based vertical skin area with its panniculus carnosus was elevated and continuously resutured back to its original position using 4-0 monofilament nylon (Ethicon, Somerville, NJ, USA) (Supplementary Fig. 1C,D)^17,20^.

### Nicotine administration

Nicotine (Sigma Chemical, St Louis, MO, USA) was given at a dose of 4 mg/kg by daily SC injections in 1 ml normal saline for 40 days before surgery and 7 days after^18^. Nicotine was injected at the site of the base of the future flap^8^. SC injections were used for slower absorption rates to overcome the high toxicity of nicotine.

### Sildenafil treatment

Sildenafil 100 mg tablets (Viagra, Pfizer Labs, NY, USA) were ground and mixed with a volume of 10 mL saline solution to obtain a simple syrup with a final concentration of 2.5 mg/mL, which was then stored in opaque bottles until use. Sildenafil was given by oral gavage through a flexible gastric tube at a dose of 20 mg/kg/day for 7 days starting from the day of operation until sacrifice^19^.

### Nitroglycerin treatment

NTG 2% ointment (Nitro-Bid, Fougera, Altana Co., Melville, NY, USA) was locally applied to form a visible coat over the flap every 8 hours starting from the day of the operation until the day of sacrifice. The ointment application was confined to the flap area^17^.

### Flap survival assessment

On the 3^rd^ and 7^th^ days after surgery, the total flap and surviving skin areas were measured. The percentage survival rate of the flap was calculated using the following equation: Percentage of skin flap survival = survived skin area/total flap area × 100^21^.

Flap survivability was assessed according to skin colour, eschar formation, and capillary refill^5^. The desired skin surface areas were measured using ImageJ software after photography and calibration of the rat using a Nikon Coolpix 4300 digital camera (Nikon Corp., Tokyo, Japan)^19^.

### Histopathological assessment

On the 7^th^ day after surgery and after euthanization of the rats by an overdose of ketamine and xylazine, the skin tissue samples were mainly collected from the transitional zones between the healthy and necrotic areas^2^. Biopsies were fixed by submersion in 10% buffered formalin and then embedded in paraffin; next, 6 μm thick sections were obtained and stained with haematoxylin and eosin (Hx&E)^22^.

### Immunohistochemical assessment

For immunohistochemical staining, sections were deparaffinized and hydrated, and blocking endogenous peroxidase activity was achieved with 3% hydrogen peroxide for 10 minutes. Blocking of nonspecific binding was performed with 1% bovine serum albumin incubation for 30 minutes. Primary antibodies against caspase-3 (Pharmingen, San Diego, CA, USA) (1:50) and proliferating cell nuclear antigen (PCNA) (1:100) (Dako Corporation, Carpinteria, CA, USA) were incubated with tissue sections overnight at 4 °C. Secondary antibodies were added for an additional 30 minutes, and then sections were incubated with horseradish peroxidase-streptavidin for another 30 minutes. The colour was developed using 3,3′-diaminobenzidine for 5 minutes, followed by haematoxylin counterstaining^23,24^.

### Morphometric analysis

The measurements were performed by only one investigator to reduce inter-observer bias. For each animal, 5 sections were investigated, and from each section, 5 different regions were observed for the microscopic morphometric analysis using ImageJ software. The Hx&E sections were evaluated for epidermal thickness at ×200 magnification^25,26^. Avoiding the follicular areas, apoptotic and proliferative indices were calculated at caspase-3 and PCNA immuno-stained sections, respectively, at a magnification of ×100, using the following equation: Apoptotic index = number of caspase 3 positive nuclei/total number of nuclei ×100

### Transmission electron microscopy

Biopsies of 1 mm^3^ size were fixed by submersion in 2.5% glutaraldehyde for 2 hours. Post-fixation was accomplished by treatment with 1% osmium tetroxide (pH 7.4) for 1 hour. Specimens were then dehydrated by alcohol at graded concentrations followed by embedding in resin. Semithin sections were prepared and stained with 1% toluidine blue and then examined under a light microscope at ×1000 magnification to detect the areas of interest. Double staining of the ultrathin sections was performed with uranyl acetate and lead citrate for subsequent examination using a JOEL transmission electron microscope (TEM) (Columbia, South Carolina, USA) at the Electron Microscope Unit, Al-Azhar University, Cairo, Egypt^27,28^.

### Statistical analysis

Data are expressed as the mean ± standard deviation (SD). Comparisons between the means of the study groups were evaluated using analysis of variance (one-way ANOVA) followed by Tukey’s *post hoc* test. A P-value of 0.05 was considered statistically significant. All statistical analyses were performed using the statistical package for the social sciences (SPSS) version 24 (SPSS, Chicago, USA)^1^.

## Results

### Subcutaneous injection of nicotine results in a model of ischemic skin flap

**Figure 1:**
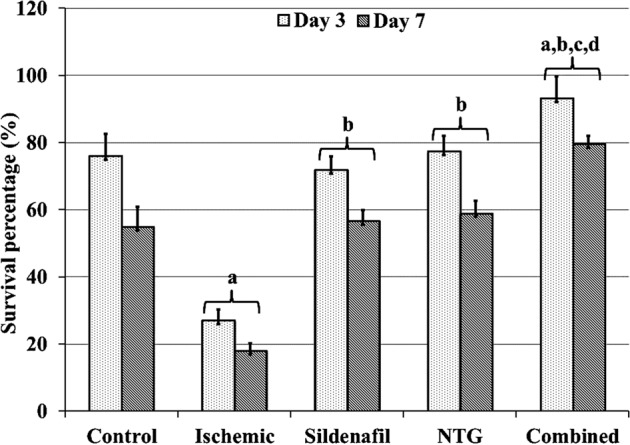
Percentages of skin flap survival at days 3 and 7 after operation of study groups.

**Figure 2:**
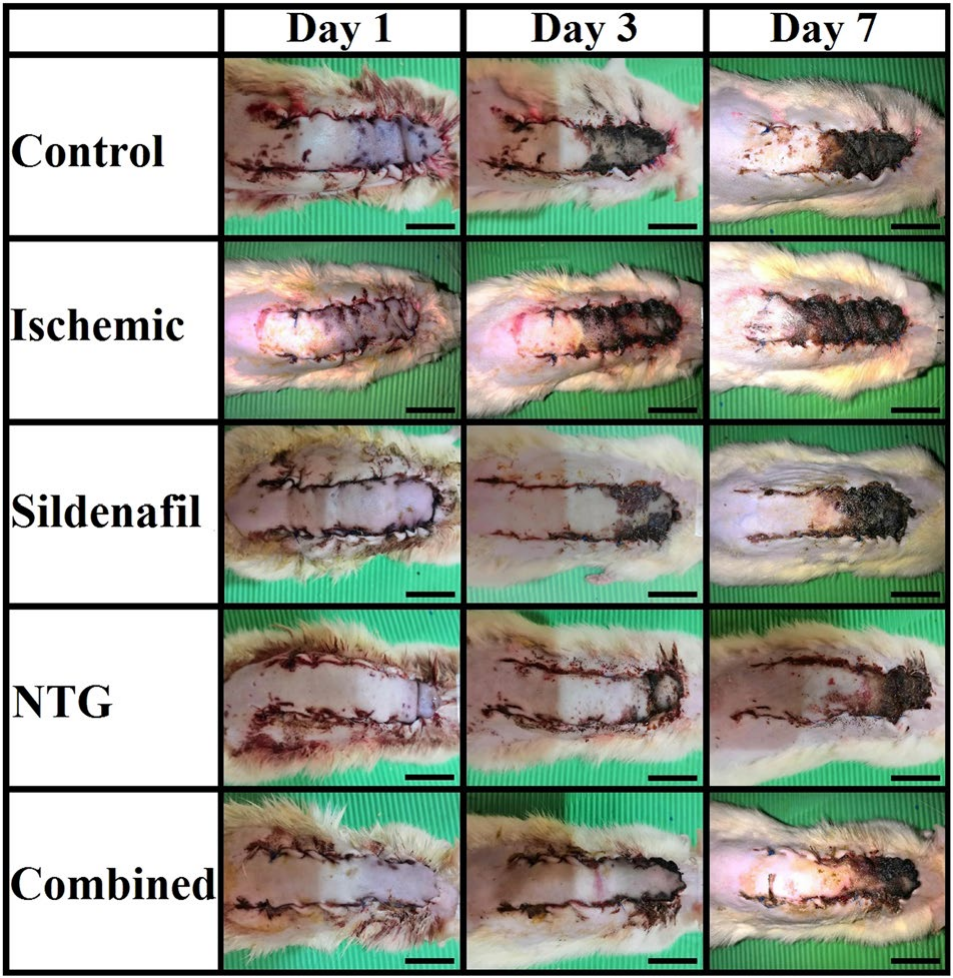
Gross appearance of skin flaps of the study groups at days 1, 3 and 7 after operation. (Scale bar = 2 cm).

The effects of flap ischemia were highly evident in rats administrated nicotine compared to the control ones based on gross, histological, immunohistochemical, morphometric and TEM analysis. In this context and regarding the percentage of skin flap survival at days 3 and 7 after the operation, a significant decrease was found in the ischaemic group compared to the control one (Fig. 1). Grossly, the ischaemic group showed a rapid progression in the process of skin necrosis in which the eschar was firm, appeared deeply coloured and started to be noticed from the postoperative day 2. In the control group, eschar formation started to be noticed on the postoperative day 3 (Fig. 2). After dissection of the flap at postoperative day 7, its deep surface showed visible blood vessels near its base that were not dilated in the control group (Supplementary Fig. 2A). In contrast, in the ischaemic group, these blood vessels were not visible and the deep surface of the flap was pale (Supplementary Fig. 2B).

The main pathological features were more obvious at the transitional area between the area of flap necrosis and the healthy skin. Supplementary Fig. 3 shows the main findings of Hx&E-stained flap sections. Ischemic group presented marked degenerative changes other than the control one e.g. thinner epidermis, increased number of keratinocytes with pyknotic nuclei and vacuolated cytoplasm, loss of demarcation between epidermal layers, and atrophied hair follicles with hardly detectable blood vessels in the dermis (Supplementary Fig. 3A,B).

Immunohistochemically, caspase-3 staining was positive in many nuclei of keratinocytes of the control group (Supplementary Fig. 4A) and affecting most of the keratinocytes nuclei in the ischaemic one (Supplementary Fig. 4B). According to PCNA immunostaining, the control group showed few nuclei within the stratum basale layer with a positive reaction (Supplementary Fig. 5A), while the epidermis of the ischaemic group was mostly negative (Supplementary Fig. 5B).

**Figure 3:**
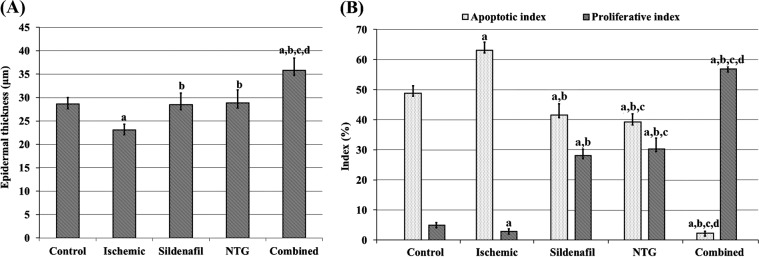
(**A**) Epidermal thickness. (**B**) Apoptotic and proliferative indices. ^a^P < 0.05 vs control group, ^b^P < 0.05 vs ischemic group, ^c^P < 0.05 vs sildenafil group and ^d^P < 0.05 vs NTG group. Statistical analysis was performed by ANOVA followed by Tukey’s *post hoc* test.

Morphometric analysis revealed a significant statistical decrease in the epidermal thickness (Fig. 3A) and proliferative index (Fig. 3B) was shown in the ischaemic group compared to the control one. On the other hand, the apoptotic index was significantly increased in the ischaemic group versus the control one (Fig. 3B).

Ultrathin sections of skin flaps of the control group showed that the keratinocytes had irregular euchromatic nuclei and scanty cytoplasm with large cytoplasmic vacuolations. In addition, the mitochondria were swollen and the rough endoplasmic reticulum was absent. On the other hand, the dermis showed absence of the blood vessels and massive irregularity of its collagen fibres with areas of collagen fibre loss. The fibroblasts in the dermis showed irregular nuclei with peripheral condensation of their chromatin, swollen mitochondria and several cytoplasmic vacuoles that enclosed electron dense materials (Supplementary Fig. 6A–C). Ischaemic group skin flap samples demonstrated that the keratinocytes had irregular nuclei, aggregations of keratohyalin granules in the cytoplasm of the granular cell, and absence of tonofilaments, desmosomes, mitochondria and rough endoplasmic reticulum. Furthermore, the dermis demonstrated absence of the blood vessels (Supplementary Fig. 6D–G).

### Combined sildenafil and NTG administration improves flap survival parameters

According to the skin flap survival percentage at postoperative days 3 and 7, there was no significant difference between the sidenafil and NTG groups. On the other hand, there was a significant increase in the flap survival rate in the combined group compared to that of the other treatment groups (Fig. 1).

Regarding to the gross examination, eschar formation started to appear at postoperative day 3 in the sildenafil group and postoperative day 4 in the NTG group. In contrast, the eschar area in the combined group had less extension than that of both the sildenafil and NTG groups, and was apparent starting on day 5 (Fig. 2). Furthermore, at the back of the dissected flaps, blood vessels were dilated and highly obvious in both the sildenafil and NTG groups, but flap dissection in the NTG group displayed mild haemorrhage that was not present in the sildenafil one (Supplementary Fig. 7A,B). This haemorrhage was profuse in the combined group and the blood vessels of the deep surface of the flap were massively dilated (Supplementary Fig. 7C).

Histopathologically and as shown in Supplementary Fig. 8, the sildenafil group had a degree of restoration of the demarcations between the epidermal layers. However, the dermal blood vessels were mostly not dilated (Supplementary Fig. 8A). Generally, the histopathological changes in the NTG group were mostly similar to that of the sildenafil one, but the NTG group showed less inflammatory cell recruitment in the dermis and more dilated subepidermal arterioles and venules (Supplementary Fig. 8B). On the other hand, the combined group exhibited a thicker epidermis than that of the sildenafil and NTG ones, minimal inflammatory cell recruitment, and numerous developed subepidermal dilated blood vessels (Supplementary Fig. 8C). The eschar areas of all study groups appeared the same, showing wide areas of coagulative necrosis of the epidermis, massive inflammatory cell infiltration in the dermis and absent dermal blood vessels (Supplementary Fig. 8D).

Caspase-3 immune staining showed that the epidermis of the sildenafil group had many scattered nuclei with a positive caspase-3 immune reaction (Supplementary Fig. 9A). On the other hand, in the NTG group there were areas with negative caspase-3 immune expression; alternating with focal areas with positive nuclear immune expression (Supplementary Fig. 9B). Nevertheless, the combined group mostly had adverse caspase-3 immune reactions in the epidermis (Supplementary Fig. 9C). Furthermore, both the sildenafil and NTG groups showed several PCNA-positive nuclei in the stratum basale layer (Supplementary Fig. 10A,B). Moreover, the deeper layers of the epidermis showed intense positive PCNA immune staining in the combined group (Supplementary Fig. 10C).

Morphometric analysis of the flap sections showed that the epidermal thickness was highly increased in the combined group compared to the other treatment groups. Moreover, there was no significant difference between the sildenafil and NTG groups (Fig. 3A). Furthermore, the apoptotic index was highly minimized in the combined group, which showed a significantly lower index percentage than the sildenafil and NTG ones. In contrast, the NTG group showed a significant decrease in the apoptotic index compared to that of the sildenafil group (Fig. 3B). Proliferative index in the combined group showed the highest values with a significant increase in PCNA expression. Nevertheless, the NTG group showed a significantly increased proliferative index compared to that of the sildenafil one (Fig. 3B).

Regarding TEM examination, the sildenafil group showed keratinocytes with regular round nuclei, swollen mitochondria, absent rough endoplasmic reticulum, and cytoplasmic vacuolations. On the other hand, the dermal capillaries were frequently observed with a normally appearing endothelium (Supplementary Fig. 11A–C). On the other hand, the NTG group showed most of the same features of the sildenafil one, however there were no blood vessels noted in the dermis (Supplementary Fig. 11D–F). Furthermore, the combined group skin flap samples showed keratinocytes with regular euchromatic nuclei and preserved rough endoplasmic reticulum arrays. The mitochondria of these keratinocytes were swollen but with preserved cristae. In the dermis, there were also well-developed arterioles and capillaries that had mostly wider lumens than those of the sildenafil group and were lined by endothelial cells having pinocytotic vesicles (Supplementary Fig. 11G–J).

## Discussion

A random-pattern flap is characterized by a lack of a defined vascular pattern. Most of its blood supply comes from its pedicle through musculocutaneous or septocutaneous perforating vessels^29^. Flap necrosis is one of the most common postoperative complications, and this restricts its length-to-width ratio to 1.5 to 2:1. Therefore, adequacy of the flap blood supply increases its survival^30^. Nevertheless, skin flaps can tolerate ischaemia for approximately 8–13 hours^31^.

To the best of our knowledge, this is the first study showing the effect of combined sildenafil and NTG on the survival of random-pattern skin flaps in rats with nicotine intake to mimic a smoking state based on gross, histological, immunohistochemical, morphometric and TEM assessments.

Nicotine is considered the principal alkaloid in tobacco and is responsible for smoking addictive properties and systemic side effects with a rapid onset of action^32–34^. In experimental models, the SC injection of nicotine can mimic heavy smoking in humans^35^. Locally, nicotine causes a significant decrease in flap blood flow and survival via producing vasoconstriction and intravascular thrombosis, in addition to the increase in platelet aggregation and levels of fibrinogen and blood viscosity. Furthermore, nicotine can cause direct endothelial cell damage, impaired cellular protein synthesis, modification of the lipid profile, and disturbed prostaglandin and thromboxane synthesis and/or release^36–39^.

In this study, nicotine SC injection in rats produced a significant decrease in flap survival percentages up to 17.8% compared to 54.7% in the control group (Fig. 1). Mauad *et al*.^18^, Eskitascioglu and Gunay^40^, and Shah *et al*.^8^ also reported that nicotine administration decreases random flap survival rate to a range between 77.7% and 50.4%. These different percentages may be due to the difference in flap size, nicotine doses used, site of nicotine injection or species of the rats, but in general nicotine showed a deleterious effect on skin flaps.

Sildenafil is a phosphodiesterase type 5 enzyme inhibitor. Inhibition of this enzyme increases the cyclic guanosine monophosphate levels inside cells and the NO concentration in the surrounding environment, which in turn leads to vascular smooth muscle relaxation, vasodilatation, inhibition of platelet aggregation, an increased blood flow, stimulation of angiogenesis and a decreased severity of inflammatory reactions^4,41,42^.

In the current study, sildenafil alone was able to decrease the extent of distal necrosis of the skin flaps. This finding was in harmony with the results of Kaya *et al*.^43^, Sarifakioglu *et al*.^44^, Tsai *et al*.^45^ and Shah *et al*.^8^ who used sildenafil also as a treatment and concluded that sildenafil is able to improve skin flap survival in a dose-dependent manner. In contrast, Figueiredo *et al*.^46^, Barral *et al*.^47^, Serin *et al*.^4^ and Ayyildiz *et al*.^11^ had revealed that sildenafil does not affect skin viability. These negative data shown by those authors for sildenafil treatment may be due to the different doses or the different routes of administration that were used. Overall, most publications have concluded that oral and IP administrations of sildenafil are more effective than local application in improving skin flap survival.

Topical NTG can induce venous and arterial vessel dilation by smooth muscle relaxation of their walls^14^. Although NTG is bioactivated into NO, there are some suggestions that its vasodilator effect is due to a NO-independent mechanism, which is evidenced in recent experimental and clinical studies showing that NO alone cannot exert all of the vasodilatory effects, in turn suggesting that additional vasoactive mechanisms are involved^48,49^. NTG also can exert its vasodilatory action through the activation of endothelial cells to synthesize prostacyclin. Prostacyclin is an effective vasodilator and inhibits platelet activation, which enhances flap survival and decreases small vascular thrombosis^14^.

In this study, locally applied NTG ointment also had a positive effect on skin flap viability. Yun *et al*.^14^ showed similar results in a human study. In contrast, Smith and Dolan^17^, and Sá *et al*.^50^ reported that NTG had no effect on skin survival after skin flap elevation in rats These different results regarding NTG treatment may be due to the variable forms of application and the different animal models of the skin flap.

Histological findings related to rats of the control group in the present study revealed the same results of Ayyildiz *et al*.^11^, Choi *et al*.^19^, and Rech *et al*.^20^ who showed that the main pathological changes in the skin flap are epidermal exfoliation, widespread neutrophil leukocyte infiltration, increased granulation tissue, increased inflammation, necrosis, and fibroblastic reaction (Supplementary Fig. 3A). In this study, these histological findings were aggravated by nicotine (Supplementary Fig. 9B) but ameliorated by sildenafil or NTG treatment (Supplementary Fig. 8A,B). Moreover, the improvement was noted in the NTG and combined groups more than in the group administered sildenafil alone (Supplementary Fig. 8). Choi *et al*.^19^ showed the same ameliorative effect of sildenafil according to their histological findings.

In this study, epidermal thickness was measured to confirm the histological findings. The epidermal thickness was maximal in the combined group (35.8 ± 2.7 μm) (Fig. 3A). Ersel *et al*.^51^ measured the normal epidermal thickness of the dorsal skin in rats, which was 40 ± 1.2 μm, and this finding indicates that, in the current study, combined, sildenafil and NTG treatment increased the epidermal thickness to approach the normal values (Fig. 3A).

Immunohistochemically, this study revealed that caspase-3 expression was decreased in groups that received sildenafil or NTG only and became highly minimized in the combined group (Supplementary Fig. 9). Furthermore, the apoptotic index was significantly decreased in the combined group compared to all other study groups (Fig. 3B). Caspase-3 is an important biochemical marker of apoptosis^52^. Apoptosis cascades can be induced during tissue ischaemia, and this induction is associated with the upregulation of Bax and downregulation of Bcl-2 in addition to increasing activity of caspase-3^53,54^. Sildenafil can induce apoptosis-protective effects via an NO synthase-dependent mechanism^55^. In addition, NTG might also interfere with apoptotic cascades through various mechanisms, including inhibition of cytochrome c release and activation of nuclear factor κ-light-chain-enhancer of activated B cells (NF-κB)^56^, which in the current study may explain why the dual administration of sildenafil and NTG had a significant effect in decreasing the apoptotic index due to the co-antiapoptotic effects of both agents with different mechanisms.

In contrast, the current study revealed that PCNA expression was increased in relation to that in the sildenafil- or NTG-only groups (Supplementary Fig. 10A,B), with a maximal increase in the combined group (Supplementary Fig. 10C), and its expression was mainly in the deeper layers of the epidermis. On the other hand, PCNA expression was decreased in the control group and became mostly negative in the ischaemic group (Supplementary Fig. 5A,B). These findings were also confirmed by calculating the proliferative index, which was significantly increased in the combined group compared to other study groups. PCNA is an active nuclear protein implicated in DNA replication, recombination, and repair, and thus increased PCNA expression has been noted during active cell proliferation^57^. This finding may explain the high PCNA expression in the sildenafil, NTG and combined groups, indicating active repair.

Currently, the TEM findings revealed that ischaemic skin flaps showed epidermal pyknosis and dermal disturbed collagen arrangement and fibroblastic abnormalities that could confirm the gross and microscopic examinations (Supplementary Fig. 6D–G). The ultrastructure of the skin flap samples revealed that the combined group had the most significant improvement effects at the levels of integrity of the keratinocytes and fibroblasts as well as the dermal collagen arrangement and vascular proliferation (Supplementary Fig. 11G–J). However, the NTG group showed better preservation of the keratinocyte and dermal integrity than the sildenafil group (Supplementary Fig. 11A–F). The sildenafil group showed better effects on capillary proliferation, which was also shown upon gross examination of the deep surfaces of the flaps (Supplementary Fig. 11A–C), which revealed the presence of haemorrhagic spots in the sildenafil group and their absence in most of the NTG group flaps (Supplementary Fig. 7A,B).

Despite its local application, NTG ointment exerts a systemic hypotensive effect^58^. Although previous publications have reported that sildenafil alone or NTG alone, as systemic vasodilator agents, could improve the viability rate of flaps, other publications suggest that most systemic vasodilators have no effect on the enhancement of skin flap survival, mostly because their systemic hypotensive effect results in decreased skin flap perfusion, leading to increased rates of necrosis^4,14,31,44,45,59,60^. Therefore, it is essential to adjust the doses, forms of application and duration of using the combination of sildenafil and NTG in patients undergoing skin flap reconstructive surgery to avoid the possible synergistic hypotensive effect of both agents on skin flaps.

## Conclusion

Gross, histological, immunohistochemical, morphometric and TEM assessments of ischaemic random-pattern skin flaps revealed that cotreatment with oral sildenafil and local NTG is more efficacious than single-agent treatment in improving skin survival. Nevertheless, more assessments of this combined treatment are required to establish the appropriate doses and duration of treatment.

## Supplementary information

Dataset 1.
