# Supplementary material for: The Effects of Sildenafil and/or Nitroglycerin on Random-pattern Skin Flaps After Nicotine Application in Rats
Source: Sci Rep. 2020 Feb 21;10:3212. doi: 10.1038/s41598-020-60128-w (PMC7035277; doi:10.1038/s41598-020-60128-w)
Supplement: Supplementary file 1 — Dataset 1. [file 41598_2020_60128_MOESM1_ESM.pdf]

The Effects of Sildenafil and/or Nitroglycerin on Random-pattern Skin Flaps After Nicotine Application in Rats

Mohamed A. Ellabban[1], Islam Omar Abdel Fattah[2], Ghada Abdel Kader[2], Omar Salah Eldeen[1], Amir E. Mehana[3], Dina M. Khodeer[4], Hossam Hosny[5], Mahmoud S. Elbasiouny[6], Suhail Masadeh[7]

[1]Plastic and Reconstructive Surgery Unit, Suez Canal University Hospitals and Medical School, Ismailia, Egypt

[2]Human Anatomy and Embryology Department, Faculty of Medicine, Suez Canal University, Ismailia, Egypt

[3]Department of Zoology, Faculty of Science, Suez Canal University, Ismailia, Egypt

[4]Department of Pharmacology and Toxicology, Faculty of Pharmacy, Suez Canal University, Ismailia, Egypt

[5]Plastic and Reconstructive Surgery Department, Cairo University, Giza, Egypt

[6]Plastic Surgery Unit National Laser Institute, Cairo University, Giza, Egypt

[7]Podiatric Surgery, UC Heath, Cincinnati, USA

Corresponding author

Email; ellabban2005@yahoo.com (M.E)

Supplementary figure

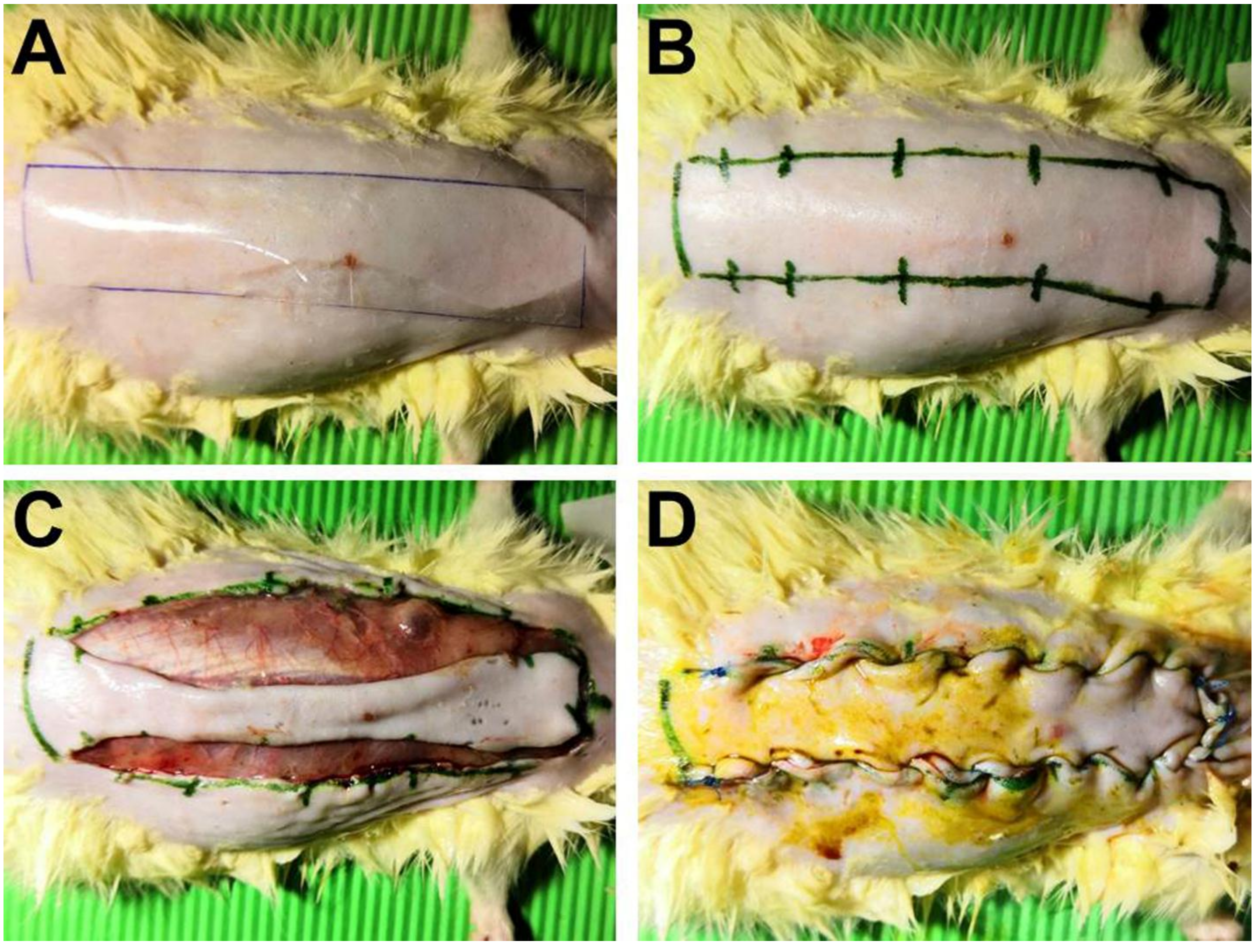

Supplementary Figure 1. Dorsal random-pattern flap procedures. **A.** A transparent template is applied to determine area of the skin flap. **B.** Drawing of the site of skin flap with indicating sites of the sutures. **C.** A skin flap after being separated from the underlying structures. **D.** Resutured skin flap.

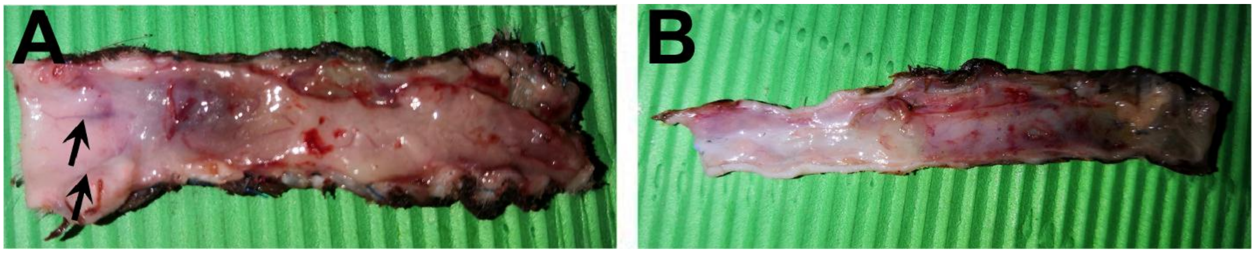

Supplementary Figure 2. The deep surfaces of skin flaps of control and ischemic groups at day 7. **A.** Control group showing visible non-dilated blood vessels (arrows). **B.** Ischemic group showing pale deep surface of the flap.

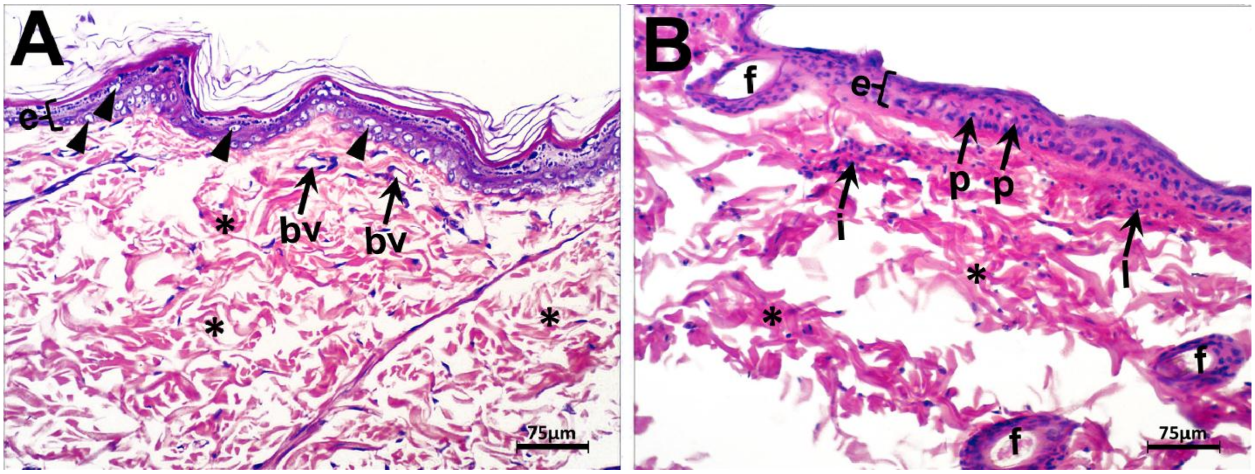

Supplementary Figure 3. Photomicrographs of sections of flap samples stained with Hx&E stain. **A.** Control group showing thin epidermis (E) with cytoplasmic vacuolations in most of its keratinocytes (arrowheads), narrow blood vessels (BV) and disorganized collagen fibers (asterisks). **B.** Ischemic group showing massive thinning of epidermis (E), absence of the demarcation between the epidermal layers, keratinocytes with pyknotic nuclei (P) and acidophilic cytoplasm, atrophied hair follicles (F), areas of inflammatory cell infiltration (I), and massively disorganized dermal collagen (asterisks). (Hx&E; ×200 – scale bar=75µm)

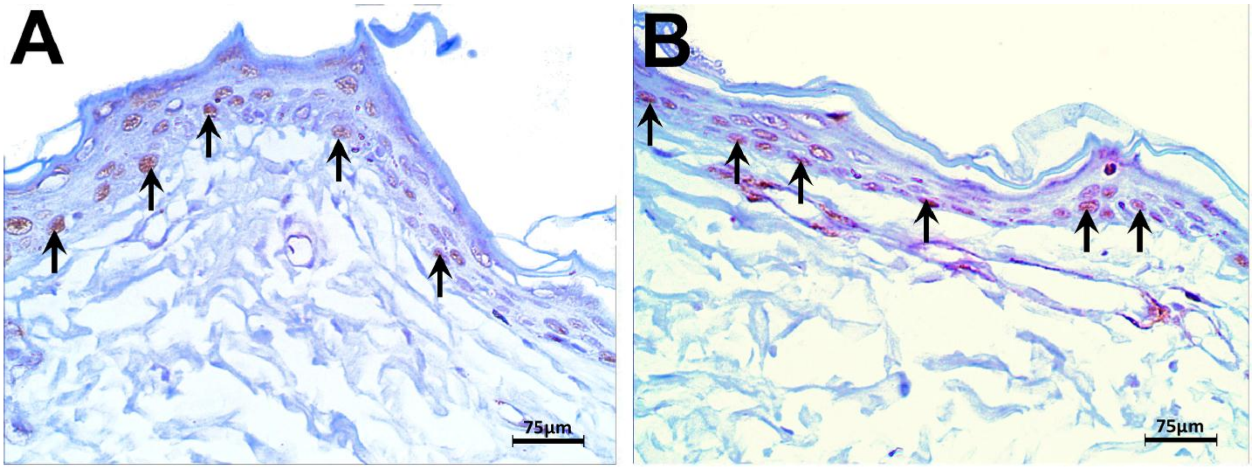

Supplementary Figure 4. Photomicrographs of sections of flap samples immunohistochemically stained with caspase-3. **A.** Control group. **B.** Ischemic group. The arrows indicate caspase-3 positive cells. (Caspase-3 immuno-stain; ×200 – scale bar=75µm)

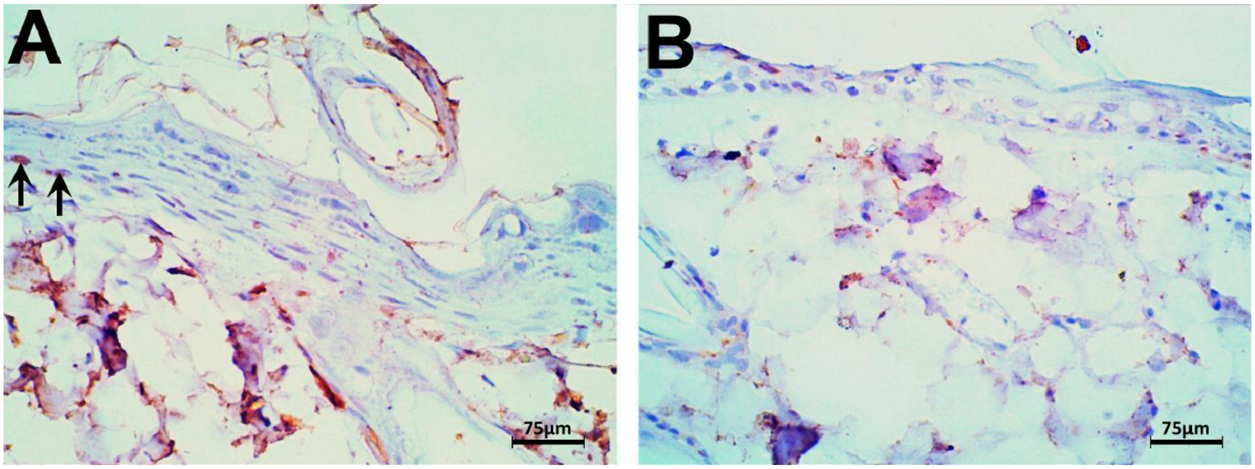

Supplementary Figure 5. Photomicrographs of sections of flap samples immunohistochemically stained with PCNA. **A.** Control group. **B.** Ischemic group. The arrows indicate PCNA-positive cells. (PCNA immuno-stain; ×200 – scale bar=75µm)

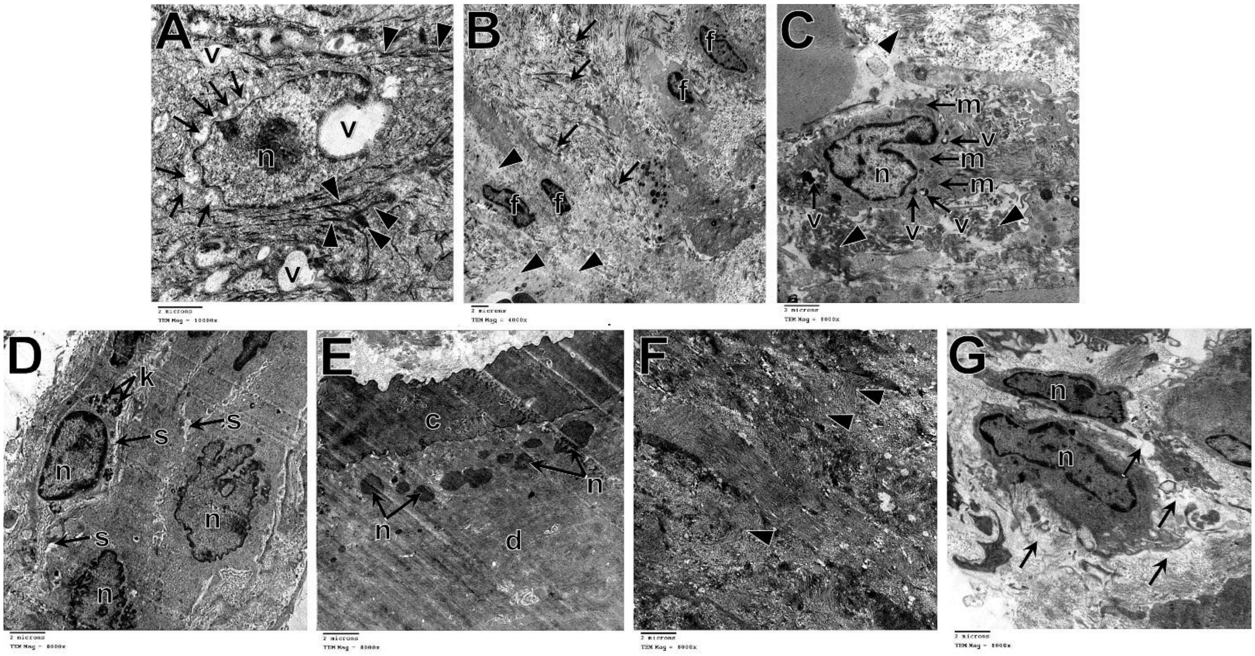

Supplementary Figure 6. Electron micrographs of skin flaps of control (A-C) and ischemic (D-G) groups. **A.** The epidermis shows a keratinocyte with an irregular euchromatic nucleus (N), relatively scanty cytoplasm, large cytoplasmic vacuolations (V), swollen mitochondria (arrows) with destructed cristae and thick bundles of tonofilaments (arrowheads). Note absence of arrays of rough endoplasmic reticulum. (TEM; ×10000 - Scale bar=2μm) **B.** The dermis shows multiple fibroblasts (F), massive irregularity of its collagen fibers (arrows) and areas of collagen fiber loss (arrowheads). Note absence of blood vessels. (TEM; ×4000 - Scale bar=2μm) **C.** The dermis shows a fibroblast with an irregular nucleus (N) with peripheral condensation of its chromatin, swollen mitochondria (m) and a number of cytoplasmic vacuoles which enclose electron dense materials (V). Note the surrounding collagen fibers which are mostly irregular and broken (arrowheads). (TEM; ×8000 - Scale bar=2μm) **D.** The epidermis shows multiple keratinocytes with irregular nuclei (N) with peripheral chromatin condensations, aggregations of keratohyalin granules in the granular cell (K) and relatively wide intercellular spaces (S). Note absence of tonofilaments, desmosomes, mitochondria and rough endoplasmic reticulum. (TEM; ×8000 -

Scale bar=2µm) **E.** The epidermis and a part of dermis showing that all keratinocytes have pyknotic nuclei (N), in addition to loss of the basement membrane separating the epidermis and the underlying dermis (D). Stratum corneum layer (C) is thickened and highly packed. Furthermore, the dermis is devoid of fibroblasts and its collagen fibers are highly condensed in their arrangement. (TEM; ×8000 - Scale bar=2µm) **F.** The dermis shows complete absence of fibroblasts and blood vessels, and massive irregularity of its collagen fibers (arrowheads) that appears highly packed together. (TEM; ×8000 - Scale bar=2µm) **G.** The dermis shows fibroblasts with peripheral condensation of chromatin in their nuclei (N) and also they are surrounded by areas of complete collagen fiber loss (arrows). Note absence of mitochondria. (TEM; ×8000 - Scale bar=2µm)

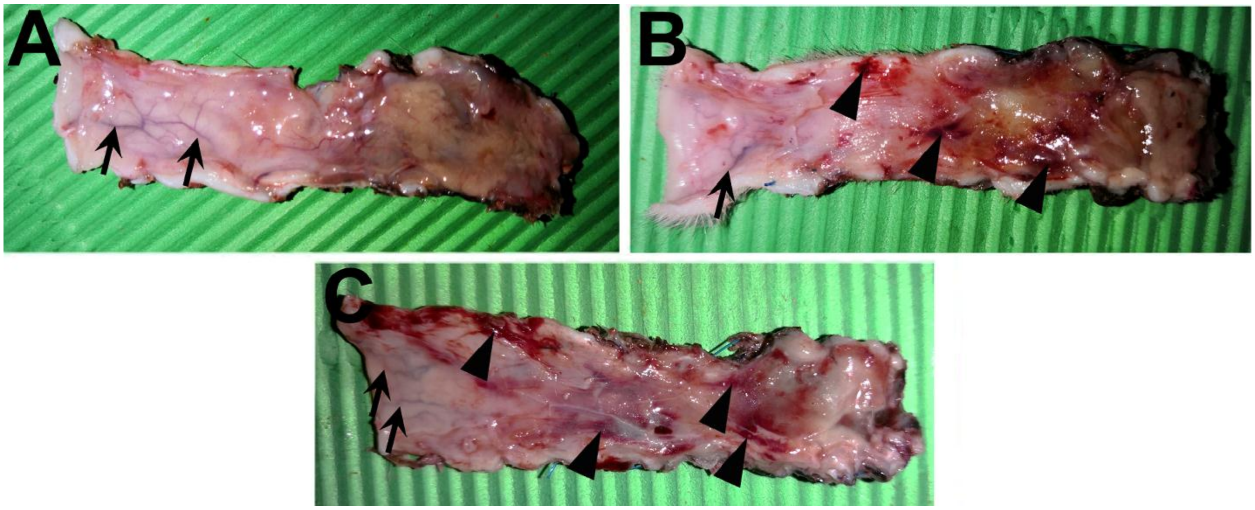

Supplementary Figure 7. The deep surfaces of skin flaps of sildenafil, NTG and combined groups at day 7. **A.** Sildenafil group showing dilated blood vessels (arrows) without hemorrhage. **B.** NTG group showing dilated blood vessels (arrow) and areas of mild hemorrhage (arrowheads). **C.** Combined group showing massively dilated blood vessels (arrows) and areas of profuse hemorrhage (arrowheads).

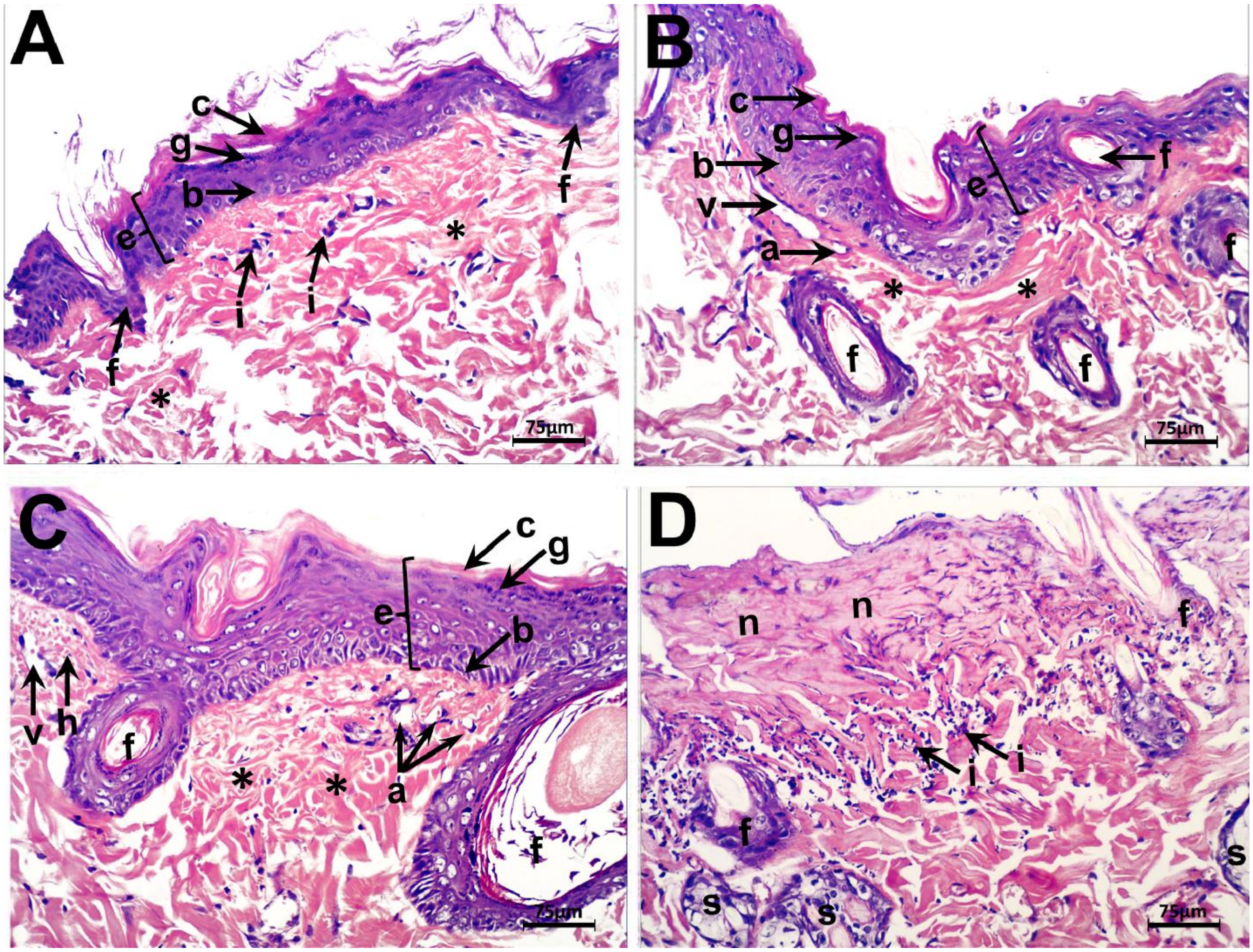

Supplementary Figure 8. Photomicrographs of sections of flap samples stained with Hx&E stain. **A.** Sildenafil group showing some regaining of the thickness of the epidermis (E), restoration of stratum basale (B), granulosum (G) and corneum (C) layers, well-developed hair follicles (F), less inflammatory cell recruitment in the dermis (I), and better organization of dermal collagen (asterisks). **B.** NTG group showing thicker epidermis (E), more numerous hair follicles (F) and better organization of dermal collagen (asterisks) than that of sildenafil group. Also, there is regaining of stratum basale (B), granulosum (G) and corneum (C) layers. The subepidermal arterioles (A) and venules (V) are dilated (asterisks). **C.** Combined group showing thick epidermis (E), several dilated subepidermal arterioles (A) and venules (V), well-developed hair follicles (F), well-organized dermal collagen (asterisks), and subepidermal hemorrhagic

areas (H). **D.** Distal necrotic zone of a skin flap showing wide areas of coagulative necrosis of the epidermis (N), massive inflammatory cell infiltration in the dermis (I), and atrophied hair follicles (F) and sebaceous glands (S). These pathological changes are the same in all study groups. (Hx&E; ×200 – scale bar=75µm)

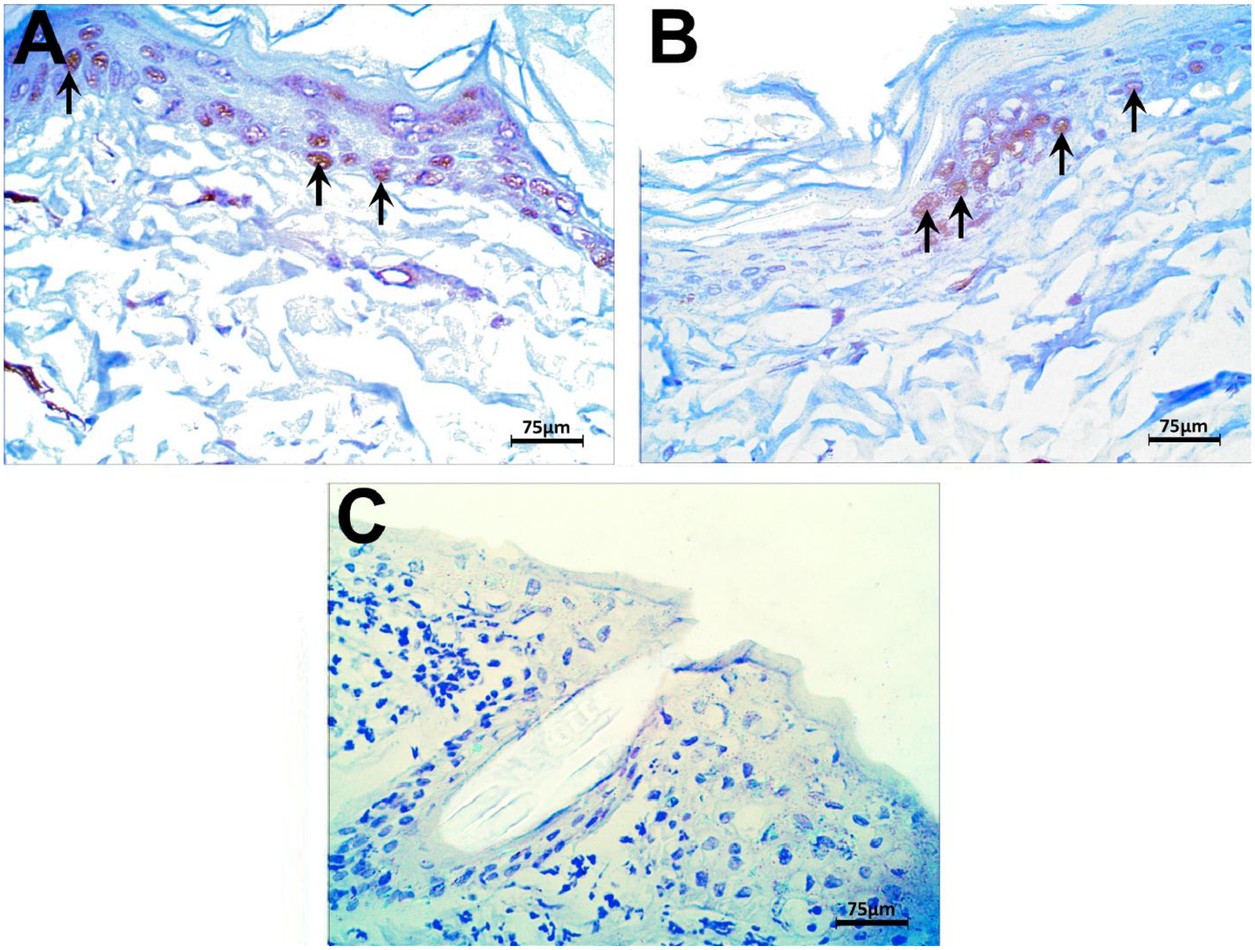

Supplementary Figure 9. Photomicrographs of sections of flap samples immunohistochemically stained with caspase-3. **A.** Sildenafil group. **B.** NTG group. **C.** Combined group. The arrows indicate caspase-3 positive cells. (Caspase-3 immuno-stain; ×200 – scale bar=75µm)

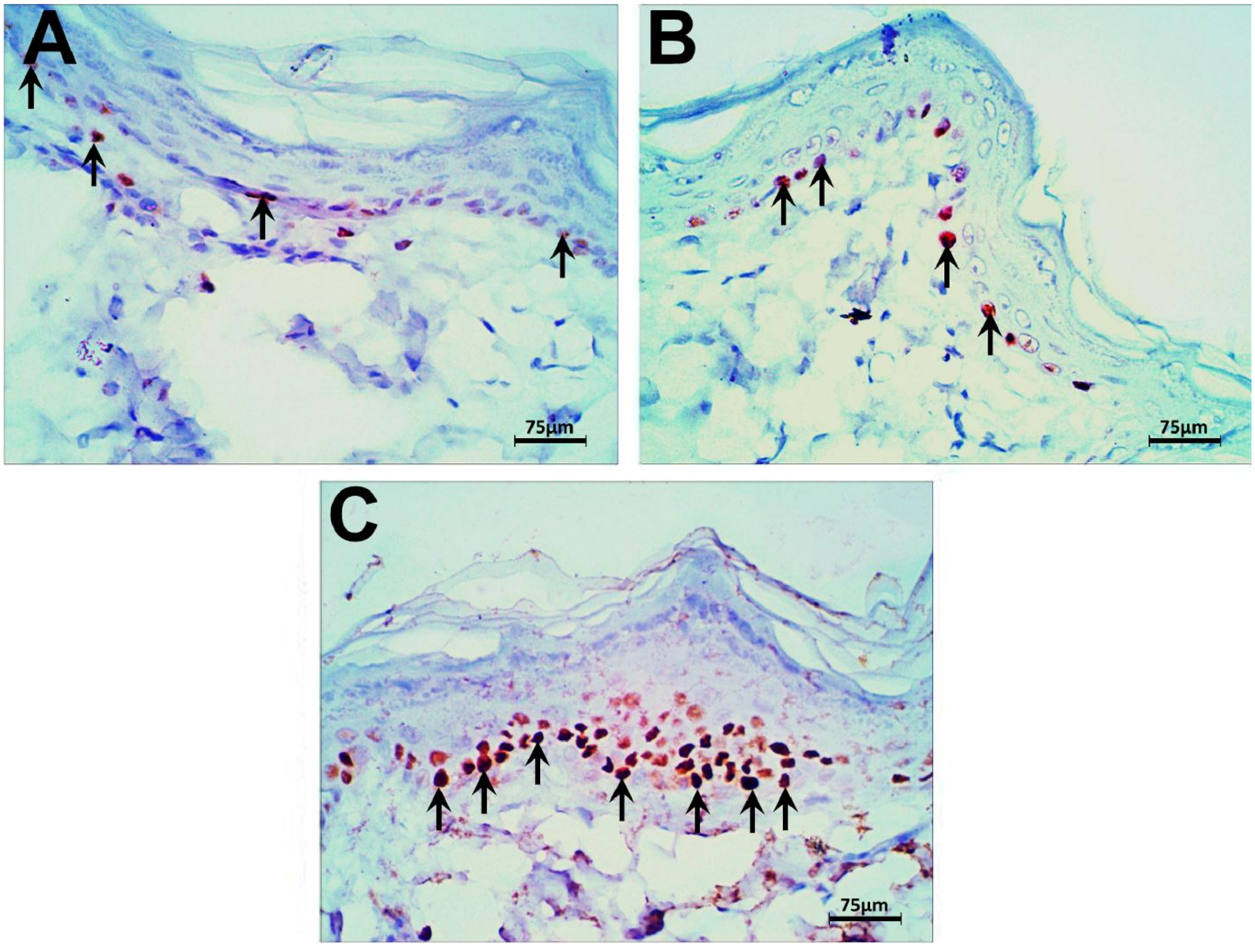

Supplementary Figure 10. Photomicrographs of sections of flap samples immunohistochemically stained with PCNA. **A.** Sildenafil group. **B.** NTG group. **C.** Combined group. The arrows indicate PCNA-positive cells. (PCNA immuno-stain; ×200 – scale bar=75μm)

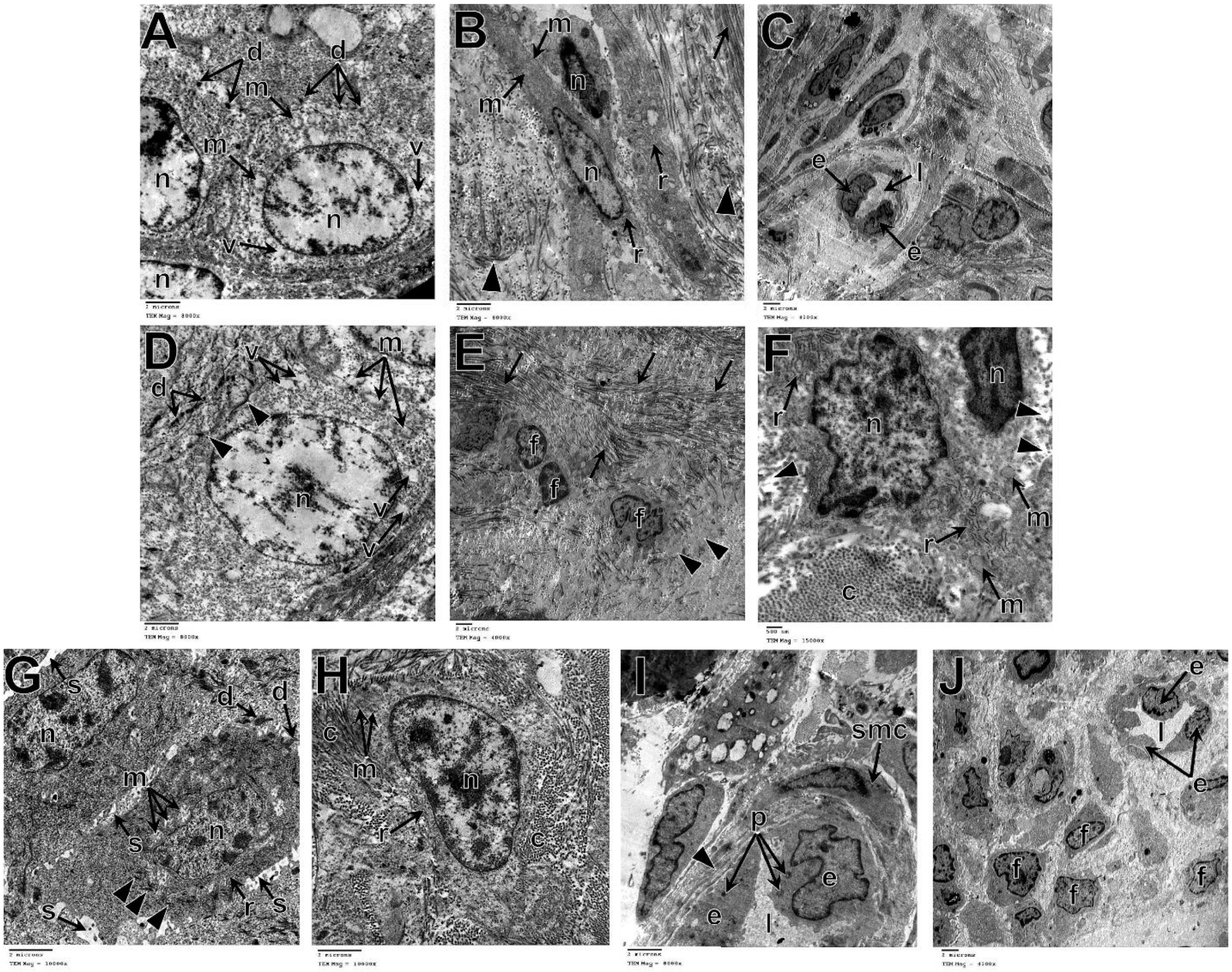

Supplementary Figure 11. Electron micrographs of skin flaps of sildenafil (A-C), NTG (D-F) and combined (G-J) groups. **A.** The epidermis shows keratinocytes with regular round nuclei (N) with loose chromatin, swollen mitochondria (M) with destructed cristae and cytoplasmic vacuolations (V) that are smaller than that of control group. Furthermore, the keratinocytes are tightly connected by desmosomes with no intercellular spaces (D). Note absence of arrays of rough endoplasmic reticulum. (TEM; ×10000 - Scale bar=2μm) **B.** The dermis shows fibroblasts with peripheral condensation of chromatin in their nuclei (N), moderately dilated arrays of rough endoplasmic reticulum (R) and normally appearing mitochondria (M). Some of the surrounding collagen fibers are regular (arrows) and others are irregular (arrowheads). (TEM; ×8000 - Scale

bar=2µm) **C.** The dermis shows a blood capillary lined by endothelial cells (E) surrounding a lumen (L). (TEM; ×4000 - Scale bar=2µm) **D.** The epidermis shows a keratinocyte with regular round nuclei (N) with loose chromatin and mitochondria (M) with destructed cristae. However, It is tightly connected to the neighboring ones by desmosomes (D) with no intercellular spaces. Note presence tonofilaments (arrowheads) and cytoplasmic vacuolations (V), despite these vacuolations are smaller than that of control group. (TEM; ×8000 - Scale bar=2µm) **E.** The dermis shows fibroblasts (F) and collagen fibers that are mostly regularly arranged (arrows); however there are areas of absent collagen fibers (arrowheads). Note absence of blood vessels. (TEM; ×4000 - Scale bar=2µm) **F.** The dermis shows fibroblasts with peripheral condensation of chromatin in their nuclei (N), dilated arrays of rough endoplasmic reticulum (R) and normally appearing mitochondria (M). Most of the surrounding collagen fibers are regular (C); however there are small areas of collagen loss (arrowheads). (TEM; ×15000 - Scale bar=0.5µm) **G.** The epidermis shows keratinocytes with mostly regular euchromatic nuclei (N) and swollen mitochondria (M); however their cristae are mostly preserved. Moreover, there are tonofilaments (arrowheads), desmosomes (D), despite there are relatively wide intercellular spaces (S). In addition, the arrays of rough endoplasmic reticulum (R) are preserved. (TEM; ×10000 - Scale bar = 2µm) **H.** The dermis shows a fibroblast with normally distributed chromatin in its nucleus (N), in addition to normal non-dilated rough endoplasmic reticulum arrays (R) and normally appearing mitochondria (M). Most of the surrounding collagen fibers are regularly arranged (C). (TEM; ×10000 - Scale bar=2µm) **I.** The dermis shows a well-developed arteriole with a lumen (L) and is lined by endothelial cells (E) that have many pinocytotic vesicles, in addition to presence of a smooth muscle cell (SMC) and elastic fibers (arrowhead) in its wall. (TEM; ×8000 - Scale bar=2µm) **J.** The dermis shows fibroblasts (F) and a blood capillary that is lined by

endothelial cells (E) that surround a lumen (L). Note the wider caliper of the capillary compared to that of sildenafil group. (TEM; ×4000 - Scale bar=2µm)
